# Supplementary material for: Analysis of PPARγ Signaling Activity in Psoriasis
Source: Int J Mol Sci. 2021 Aug 10;22(16):8603. doi: 10.3390/ijms22168603 (PMC8395241; doi:10.3390/ijms22168603)
Supplement: Supplementary file 1 [file ijms-22-08603-s001.zip › Supplemental materials_Analysis of PPARg signaling activity in psoriasis/Pathway models/Models images and html files/PPARG inhibits phenotypes assotiated with psoriasis/149094.html]

arteriogenesis


# Cell Process arteriogenesis

|  |  |
| --- | --- |
| URN | urn:agi-gocellproc:0048844 |
| Total Entities | 0 |
| Connectivity | 687 |
| Name | arteriogenesis |

---

|  |  |
| --- | --- |
| Pathway | PPARG inhibits phenotypes assotiated with psoriasis |
|  | efects selected |

---

|  |  |
| --- | --- |
| MedScan ID | 4048844 |

---

|  |  |
| --- | --- |
| Alias | arterial formation |
|  | arterial adventitia vasculature |
|  | arteriogenesis |
|  | arterial revascularization |
|  | arterial re-vascularizations |
|  | aorta vasculature |
|  | arterial adventitia revascularization |
|  | aorta re-vascularization |
|  | artery formations |
|  | arterial morphogeneses |
|  | aortic revascularization |
|  | artery morphogeneses |
|  | arterial growth |
|  | artery re-vascularizations |
|  | aorta growth |
|  | arterial vasculatures |
|  | aortic revascularizations |
|  | arterial formations |
|  | aortic vasculature |
|  | arterial adventitia growth |
|  | aorta re-vascularizations |
|  | aortic re-vascularizations |
|  | artery morphogenesis |
|  | aortic growth |
|  | arterial re-vascularization |
|  | arterial morphogenesis |
|  | aorta revascularization |
|  | artery growth |
|  | artery revascularizations |
|  | arteriogeneses |
|  | artery vasculature |
|  | aortic re-vascularization |
|  | arterial vasculature |
|  | artery formation |
|  | aorta revascularizations |
|  | growth of the aorta |
|  | artery revascularization |
|  | artery re-vascularization |
|  | arterial revascularizations |
|  | aorta re-vascularisations |
|  | aorta revascularisations |
|  | aortic re-vascularisation |
|  | arterial re-vascularisations |
|  | arterial revascularisations |
|  | arterial revascularisation |
|  | artery revascularisations |
|  | aorta re-vascularisation |
|  | aorta revascularisation |
|  | aortic re-vascularisations |
|  | aortic revascularisations |
|  | aortic revascularisation |
|  | arterial re-vascularisation |
|  | artery re-vascularisations |
|  | artery re-vascularisation |
|  | artery revascularisation |

---

|  |  |
| --- | --- |
| GO ID | 0048844 |

---
